# Adaptation and Integration of Psychosocial Stimulation, Maternal Mental Health and Nutritional Interventions for Pregnant and Lactating Women in Rural Bangladesh

**DOI:** 10.3390/ijerph17176233

**Published:** 2020-08-27

**Authors:** Fahmida Akter, Mahbubur Rahman, Helen O. Pitchik, Peter J. Winch, Lia C. H. Fernald, Tarique Mohammad Nurul Huda, Tania Jahir, Ruhul Amin, Jyoti Bhushan Das, Khobair Hossain, Abul Kashem Shoab, Rizwana Khan, Farzana Yeasmin, Jesmin Sultana, Stephen P. Luby, Fahmida Tofail

**Affiliations:** 1 Infectious Diseases Division, International Centre for Diarrhoeal Disease Research, Bangladesh (icddr,b), Mohakhali, Dhaka 1212, Bangladesh; mahbubr@icddrb.org (M.R.); tarique.huda@icddrb.org (T.M.N.H.); tania.jahir@icddrb.org (T.J.); ruhul.amin@icddrb.org (R.A.); jyoti.das@icddrb.org (J.B.D.); khobair.hossain@icddrb.org (K.H.); akmshoab@icddrb.org (A.K.S.); rizwana.khan@icddrb.org (R.K.); fyeasmin@icddrb.org (F.Y.); jesmin.sultana@icddrb.org (J.S.); 2 Division of Epidemiology, School of Public Health, University of California, Berkeley, CA 94720, USA; hpitchik@berkeley.edu; 3 Department of International Health, John Hopkins Bloomberg School of Public Health, Johns Hopkins University, Baltimore, MD 21205, USA; pwinch@jhu.edu; 4 Division of Community Health Sciences, School of Public Health, University of California, Berkeley, CA 94720, USA; fernald@berkeley.edu; 5 Division of Infectious Diseases and Geographic Medicine, Stanford University, Stanford, CA 94305, USA; sluby@stanford.edu; 6 Nutrition and Clinical Services Division, International Centre for Diarrhoeal Disease Research, Bangladesh (icddr,b), Dhaka 1212, Bangladesh; ftofail@icddrb.org

**Keywords:** psychosocial stimulation, maternal mental health, nutrition, early childhood development, integrated intervention, adaptation

## Abstract

Supporting caregivers’ mental wellbeing and ability to provide psychosocial stimulation may promote early childhood development. This paper describes the systematic approach of developing an integrated stimulation intervention, identifying the feasibility and challenges faced throughout the period. We developed an integrated curriculum by culturally adapting three interventions (Reach Up, Thinking Healthy, and general nutrition advice) and piloted this curriculum (Mar–April 2017) in courtyard groups sessions and individual home visits with pregnant women (*n* = 11) and lactating mothers (of children <24 months) (*n* = 29). We conducted qualitative interviews with the participants (*n* = 8) and the community health workers who delivered the intervention (*n* = 2). Most participants reported willingness to attend the sessions if extended for 1 year, and recommended additional visual cues and interactive role-play activities to make the sessions more engaging. Participants and community health workers found it difficult to understand the concept of “unhealthy thoughts” in the curriculum. This component was then revised to include a simplified behavior-focused story. Community health workers reported difficulty balancing the required content of the integrated curriculum but were able to manage after the contents were reduced. The revised intervention is likely feasible to deliver to a group of pregnant and lactating mothers in a low-resource setting.

## Key Messages

- Extending the scope of early child development intervention by including pregnancy and the post-childbirth periods, as well as maternal mental health is feasible in a low-resource setting;
- Development of such an integrated intervention requires a systematic process for the adaptation and feasibility testing;
- The three adaptation phases: situation analysis, intervention pilot, and identification of facilitators and barriers through participant feedback allow for productive curriculum revision;
- Inclusion of a greater number of visual cues, interactive role-play activities, simplified mental health contents, motivational meetings with family members, and tailoring of intervention contents to the age groups present improved intervention acceptability and ease of delivery;
- A structured adaptation process may allow for rapid development of a feasible intervention in a new population.

## 1. Background

Globally, around 250 million children under five years of age are at risk of not meeting their optimal developmental potential [1]. The first 1000 days, starting at conception, and continuing through a child’s first two years of life, represent a vulnerable period when both positive and negative experiences can exert a long-term impact on brain development [2,3]. Maternal and child nutrition, responsive parenting with psychosocial stimulation, and maternal mental health are important influences on early childhood development outcomes [2,4,5].

During pregnancy, poor nutrition and maternal depression affect child development outcomes [6,7]. A meta-analysis showed a significant association between maternal diet during pregnancy and child developmental outcomes, with the strongest impact on socio-emotional and cognitive functioning of children [6]. Maternal mental health including depressive disorder during pregnancy is associated with the structural changes in the prefrontal cortex as well as the frontal and inferior temporal regions of children’s brains [8,9], and related to poor child development outcomes in low- and middle- income countries worldwide [7].

After birth, maternal mental health, child health and nutrition, early learning opportunities, and the quality of mother-infant interaction significantly influence the language, social-emotional, and cognitive development of children [2,10,11,12,13,14]. Effective caregiver support programs can improve the quality of early care of children, the home environment, caregiver knowledge of early childhood development, and developmental outcomes [15,16]. Given the many components of an enriching and supportive environment for child development, there are increasing calls for interventions integrating curricula for multiple risk factors which co-occur during the crucial window of the first 1000 days of life [17]. Several studies in Bangladesh have shown that integrated psychosocial stimulation and nutrition interventions during early childhood improve early development for undernourished, anemic, and non-anemic children [18,19,20,21]. These studies show consistent benefits of early childhood development interventions that begin after birth, but have not addressed women during pregnancy or included a focus on maternal mental health.

Given many exposures to factors affecting health and development during pregnancy and soon after childbirth, children may not reach their optimum development. Child development interventions that start from early pregnancy and include maternal mental health components may have large impacts on child developmental outcomes. These interventions would need to address factors affecting early childhood development and be readily understandable, adapted to the local context, and easy to deliver to the target population. Developing such an integrated intervention requires systematic adaptation strategies [22,23,24] to increase participants’ adherence to the behavior recommendations. Therefore, in this study we aimed to develop an integrated psychosocial stimulation intervention to promote early childhood development that targets both pregnant and lactating mothers. We also aimed to explore the feasibility, acceptability, and barriers to implementing this integrated intervention by trained Community Health Workers (CHWs) in a low-resource setting.

## 2. Methods

### 2.1. Study Site and Design

The study was conducted in two rural villages, Adampur and Chorbetal in Kishoreganj district, Bangladesh. We applied a participatory research methodology and followed the stages developed by Goldstein et al. [24] to integrate and adapt multiple evidence-based interventions. Across three different phases, we incorporated feedback from community members, participants, and CHWs (Table 1).

### 2.2. Adaptation Phases of Integrated Intervention

#### 2.2.1. Phase 1—Identify Core Components and Structure of Intervention

The Reach-Up curriculum for early childhood stimulation was the base manual for adaptation [25,26]. The curriculum was initially developed to be delivered on a weekly basis, and designed to teach mothers how to play with home-made toys and picture books and interact with their children in a way to promote their development. Mothers were encouraged for responsive parenting by addressing their child’s interest and vocalizations with praise and positive feed-back. The activities of the curriculum were age specific, developmentally appropriate and ordered with increasing difficulty level. Emphasis was specifically given to make each session interactive and enjoyable. The curriculum was culturally adapted for Bangladesh and was simplified later for fortnightly sessions [21]. This curriculum was originally developed for children aged 6–48 months, and we expanded the intervention contents to include pregnant women and mothers of children under 6 months old. To develop these additional materials, we conducted a situation analysis in two villages through two small group discussions (guidelines: Annexure A and B), one with pregnant women (*n* = 9), and another with mothers of children <24 months old (*n* = 9). The participants of the group discussions were selected with the help of the chairman of the respective villages.

**Figure 1:**
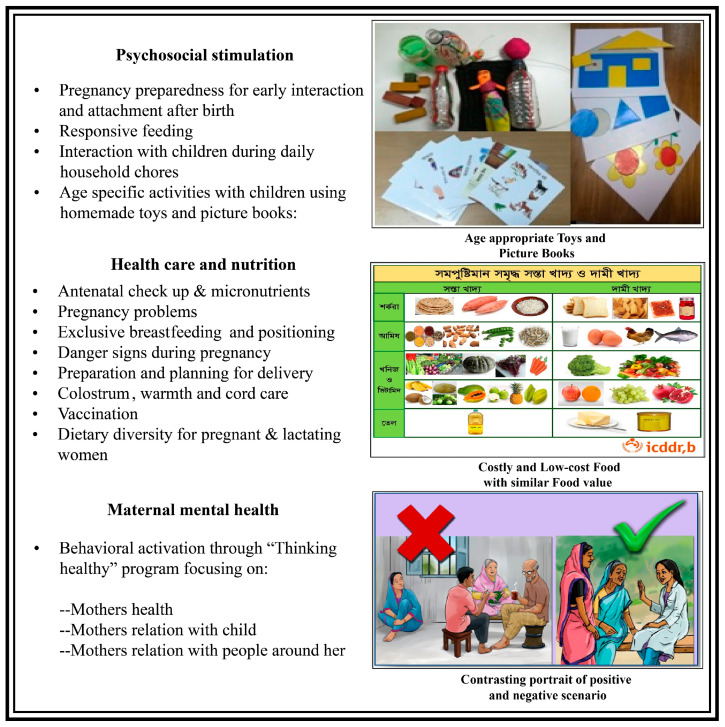
Integrated intervention components.

Following the situation analysis, we incorporated additional components into the child stimulation curriculum to address mental health and nutrition (Figure 1). For mental health, we adapted and pre-tested the Thinking Healthy intervention with culturally relevant pictures and stories that could be delivered in a short amount of time along with other integrated session contents. The original Thinking Healthy Program was mainly based on cognitive behavioral therapy and behavioral activation to assist mothers in changing their unproductive thoughts and to improve self-care, child care, and relationships with people in the community. It aimed to capture mothers’ attention toward a sequence of pictures depicting a scenario of negative thoughts-behavior-consequences and how to alter the cycle into positive thoughts-behavior and consequences. The focus was to help mothers identify their own negative behaviors and alter them into positive behaviors, and practice this in real life. The Thinking Healthy intervention previously has shown positive impacts on management of maternal depressive symptoms in rural Pakistan [27] and in Bangladesh [28]. To address nutrition and health care, we incorporated general nutritional advice and behavioral recommendations on prevention of complications for pregnant women and lactating mothers and their young children, which was obtained from the general nutritional curriculum endorsed by the Government of Bangladesh [29,30].

#### 2.2.2. Phase 2—Pilot Initial Revisions of Intervention

The aim of the pilot was to examine the feasibility of recruitment, training, and delivery of CHW-led group and individual sessions. In order to recruit the CHWs, our field staff distributed a job circular for CHW candidates in the two target villages. We then selected four local women based on interviews and a written test on general knowledge about child development, nutrition, and problem-solving ability. The four selected candidates attended 6 days of residential training on the integrated intervention curriculum. The training methods included participatory discussions, demonstrations of the play activities, role playing, and practice conducting sessions with real mothers. Based on the performance of the CHWs in both the theoretical discussions and session practice, we made a final selection of two CHWs from two villages. A household listing was conducted by the two CHWs in their respective villages. The inclusion criteria were, (i) pregnant women in their 1st or 2nd trimester and (ii) mothers of children <24 months of age. Based on the household listing, 20 participants were randomly selected from each village. Because of the unavailability of additional pregnant women, the number of enrolled pregnant women (*n* = 11) were less than the number of lactating mothers (*n* = 29). It is already well established that child psychosocial stimulation intervention is effective, specifically when it is given through one-to-one contact in individual settings [18,19,20]. But individual level service delivery is labor intense, expensive, and not feasible for scale up. So as an alternative option we evaluated the feasibility of a less intense, integrated group-based ECD intervention for future scalability, and a mixed option combining group session and more established individual sessions alternatively. Therefore, the pilot intervention sessions were delivered every two weeks over a two-month period in either courtyard group meetings (group arm, *n* = 20) or a combination of alternating individual and group meetings (combined arm, *n* = 20). Each group meeting had 5–6 participants and session providers took attendance during each session. In the group arm, a total of four detailed group sessions were delivered. In the combined arm, group meetings were short follow-up sessions that occurred between detailed individual sessions at home, with a total of two individual and two follow-up group sessions in this arm. For fidelity of the intervention delivered, the senior psychologist in the team directly observed 25% of the total sessions in both the arm. The focus was to increase the quality of sessions and the major points included rapport build up with participants, simplicity of language of discussion, interactive participation, time management, making the session enjoyable, retaining the information, and ensuring the practice at home through daily activities. Any weaknesses/issues related to optimum session delivery were discussed every week through meeting with full team.

#### 2.2.3. Phase 3—Identify Feedback from Participants and CHWs

Following the pilot sessions the research team conducted qualitative interviews (*n* = 10; 2 with CHWs, and 8 with intervention participants) using semi-structured interview guides (Annexure-C and D). The participants were selected based on an assessment conducted by the CHWs after each group session. CHWs assessed participant engagement and categorized it into high or low engagement. The participant sample consisted of four participants from each of these categories, two pregnant, and two lactating mothers in each group.

The interviews were designed to determine the participants’ and CHWs’ responses to the intervention content and delivery, and identify areas of further modification. The curriculum was then refined after incorporating this feedback and suggestions.

### 2.3. Ethical Approval

The study team collected written informed consent from all participants before data collection. This study was approved by the Institutional Review Board of icddr,b and by the Committee for the Protection of Human Subjects at the University of California, Davis.

### 2.4. Data Analysis

We conducted content analysis of the qualitative data. The team conducted systematic debriefings immediately following each group discussion and individual interview by a member of the research team who intensively observed the interview. It was similar to the peer debriefing to explore implicit aspects of the interviews. All the data were collected in the native language (Bangla), audio recorded, and translated into English during the debriefing process. Deductive codes generated before data collection based on the intervention activities and desired outcomes were first applied. Inductive coding of the facilitators and barriers of the intervention was then undertaken manually by the researcher team. A sub-set of co-authors participated in a goal-oriented discussion on the coded data, categorized the codes to generate themes, and compared them across participant groups. Based on thematic analysis, the systematic debriefing, and field notes, we identified the major adaptation requirements of the intervention during both the situation analysis phase and following the pilot intervention. Systematic debriefing has been carried out in several previous studies and considered an important process to gain immediate insight into the data, improve learning [31,32] and generate findings for immediate dissemination [33].

## 3. Results

### 3.1. Phase 1: Identified Intervention Components and Structure

The group discussion with pregnant women revealed large knowledge gaps about pregnancy, including danger signs, and the importance of maternal mental health and early interaction with children during daily activities (Table 2). Moreover, community members had alternative understandings of how to behave during pregnancy, including forbidding women from eating certain foods including cucumber, turmeric, and coconut because of concerns that these might have negative impacts on the child. Mood swings during pregnancy were believed to be ordinary and no additional effort was taken for mental wellbeing. Such alternative understandings and practices indicated the need to add a behavior modification and activation process using an adapted version of the Thinking Healthy approach.

During the group discussion with lactating mothers, participants mentioned that they find it difficult to practice the suggested activities with children because of the heavy workload at home. These findings led to the emphasis on incorporating stimulating activities during daily child-focused activities including bathing, feeding, dressing, and breastfeeding, as opposed to an addition to the existing workload. In response to these knowledge gaps, we developed an integrated curriculum to be tested in the field.

### 3.2. Phase 2: Feasibility of Implementing the Initial Revision of Intervention (Pilot Trial)

In February 2017, 40 participants were enrolled in the 2-month pilot trial, 4 of whom dropped out before the end of the intervention (2 pregnant women and 2 lactating women). A total of 36 participants (9 pregnant and 27 lactating mothers) completed the 2 month-long intervention (Table 3).

Average session attendance was 95% (*n* = 19 out of 20) in the combined arm, and 80% (*n* = 16 out of 20) of the group arm. The length of sessions in the group arm was around 40–50 min and in the combined arm, individual sessions were 20–30 min long, and group follow-up sessions were 15 min long. During this phase, the study team faced multiple barriers during training and implementation (Table 4).

The lessons learned in different phases reflected both the cultural context and intensity of the intervention curriculum, led the team to adapt several mitigation strategies. For example, during the situation analysis phase, large knowledge gaps were identified on multiple areas and it was difficult to incorporate the most essential information from the identified knowledge gaps while keeping the intervention sessions simple and short. Recruitment of the CHWs was challenging because we did not find enough candidates who had completed secondary school, and we had to relax the education criteria of completion of secondary school. The integrated curriculum demanded a residential training but the CHW candidates were not allowed by the family members to attend a residential training of long durations. Therefore, it was required to conduct the training on the integrated intervention within a limited time period. Further, we had to allow one family member to accompany the training participants and this sometimes created a distraction for the trainees. During the pilot phase, the team observed that some of the sessions were not interactive and the session duration was prolonged because the CHWs struggled to identify the key behavior recommendations to deliver from detailed curriculum and also to identify the closing remarks in the session. CHWs managed to retain mothers’ attention throughout session by reinforcing communication regarding the benefit of child development later in life. Further, family members of some of the study participants began to interfere with regular session attendance in the second month of the intervention, resulting in some dropouts. Some husbands and the mother-in laws were not motivated to allow the mothers participate in the session. In some cases, the CHWs were able to convince family members through one-on-one motivational meetings, explaining the long-term benefits of the intervention on growth and development of children, and how this can affect their performances in schools and adult professional life.

### 3.3. Phase 3: Participants’ Feedback after Intervention and Final Revision

The qualitative feedback of all the participants was categorized in respect to intervention contents, delivery, and participation. The characteristics of each participant are described in Table 5.

### 3.4. Intervention Content

Almost all participants (*n* = 7) had a favorable impression of the nutrition behavioral recommendations. The concept of early stimulation was new for the participants and all the lactating mothers liked this idea, though only one pregnant woman reported interest in the stimulation activities. Both pregnant women and lactating mothers reported acting on the behavioral recommendations that they learned in the session: some of the pregnant women (*n* = 2 of 4) reported attending an antenatal check-up; some of the lactating women (*n* = 3 of 4) reported participating in child-stimulating activities using utensils made of mud, flowers made from paper to teach the concept of color, and shakers to attract the child and watch the eye movement. One lactating mother expressed:

*Before attending the session, I used to give toys to my baby, and she played alone… Now I have learned from the session… I followed this, and I have observed the benefit. Now my 5 month old daughter can make noise, pay attention to my sounds, and look at me when I call her by name (IDI-5).*

Some of the participants also reported that they rest more (*n* = 4) and ate more nutritious food (*n* = 5) to improve their health and the development of their child, which they were unaware of before the intervention. However, one mother’s ability to follow the recommended nutrition guidelines was hindered by specific cultural norms. For example, one pregnant mother explained:

*I cannot eat between the afternoon (Asar) and evening (Maghrib) prayers, as my 27-day-old child died last year during this time period. People say that my dead son won’t get food if I take food during that time. So I will maintain this until my death (IDI-3).*

According to both CHWs, the adapted Thinking Healthy approach was more difficult for mothers to understand when compared to other components. One CHW mentioned:

*Mothers can’t understand the thinking healthy story appropriately by the pictures only. It is difficult for us also (KII-1).*

A few mothers (*n* = 3) reported behavioral activation to reduce anxiety and think productively after the adapted Thinking Healthy component and reported that it allowed them to handle their children with more patience.

For further refinement, pregnant mothers suggested including more information about pregnancy complications and solutions (*n* = 3 of 4) and both pregnant and lactating mothers recommended to include more audio-visual materials (*n* = 4) as a channel of communication during the sessions. One mother stated:

*More visual things will help us to understand more. Videos are very helpful to memorize. Drama will be interesting (IDI-4).*

### 3.5. Intervention Delivery and Participation

Mothers (*n* = 5) were satisfied with the session duration of around 50 min and almost all the mothers (*n* = 7) reported that they would be able to participate in the intervention if it extended for one year. However, some pregnant women (*n* = 2 of 4) faced discomfort while sitting in the same position for long time and reported feeling dizzy during the sessions. One CHW mentioned:

*Some pregnant women felt uneasy to sit on the bed or floor. So, they sat on the chair. They also cannot sit for long. Then I allowed them to move for a while (KII-1).*

Both CHWs also mentioned some barriers to disclose sensitive issues in the group meetings, including feminine problems, and discussing participant’s own unhealthy thinking or unproductive behaviors with the group to mobilize the Thinking Healthy process:

*Sometimes they didn’t want to share some feminine problems like danger signs if someone else was present in the session (KII-1).*

*It was hard to make them understand about the thinking healthy component and to identify their unhealthy thoughts. Who want to reveal own unproductive behavior in front of others? (KII-2)*

Acceptance of the program by husbands and neighbors was mixed. A few mothers (*n* = 3) reported that their husband and neighbors discouraged them from attending the sessions as there was no monetary benefits, while many mothers (*n* = 6) stated that their mother-in-law and other family members assisted with the household chores, which enabled the mothers to participate in the session. As stated by one mother:

*If I could not finish my work, and the session is about to begin, my mother-in-law does the remaining work and let me go for the session (IDI-6).*

In order to ensure smooth participation in the session, some mothers (*n* = 4) proposed involving husbands and neighbors in the session so that they could be aware of the program.

The key findings of the intervention contents and delivery have been described under the main themes that emerged as potential facilitators and barriers to intervention success (Table 6).

### 3.6. Final Adaptation of Integrated Intervention

In response to the feedback from the participants and CHWs, the content and delivery strategies of the intervention were further refined: (1) An introductory session that emphasizes the importance of early stimulation during the first 3 years of life was included; (2) visual cues and interactive role-play activities were included to better demonstrate concepts and to make the sessions more interactive and interesting; (3) intervention content was divided into five age groups in order to enable delivery of intervention content relevant to participants present in each session (the groups are: pregnant women and children with 0–5, 6–11, 12–18, and 19–24 months old); (4) the adapted Thinking Healthy component for maternal mental health was simplified and included a story telling approach, which depicted a contrasting portrayal of negative and positive behaviors. The original manual included six positive and negative portraits of “Thoughts-Action-Consequences” that was altered with the behavior-focused story of two contrasting life scenarios.

## 4. Discussion

In this study, we adapted and integrated three distinct intervention programs (psychosocial stimulation for children, maternal mental health, and health and nutritional advice) into a comprehensive curriculum and piloted it to identify the feasibility of the intervention contents and delivery strategy. We found that the integrated intervention was acceptable to the mothers and deliverable by the trained CHWs following ongoing modifications in different adaptation phases: situation analysis, two-month pilot, and the qualitative phase of identifying feedback from the intervention participants and CHWs. We also found that the mothers similarly adhered to both type sessions (group and combined) and retained the information for practice at home.

During the situation analysis several gaps in knowledge and practices were identified in the community, including knowledge about health during pregnancy, early stimulation for children less than 6 months of age, childcare practices during daily household activities, and strategies to improve maternal mental health. Including contents to address these gaps made the curriculum lengthy and complex. Long sessions resulted in less interest and participation, and this was observed during session implementation. Addressing these barriers required simplifying contents and streamlining delivery strategies while emphasizing age and context-specific information relevant to those who attended. In addition, we found motivating and engaging the family members played an important role in encouraging the mothers to participate in the sessions. We assumed that the community would be more motivated if the mothers are engaged in a leadership role by rotation to mobilize the group-meetings and ensure everyone’s regular participation. The mothers might be interested to continue this leadership role as this might also enhance their status in the community. We also realized that demand-based extra time for pregnant mothers for specific problems after each session might encourage them to stay longer.

The qualitative assessment following the implementation revealed mothers’ compliance with some of the behavior recommendations including increased interaction with children, more ANC visits and more health care activities including ensuring proper rest and nutrition, which supports the acceptability on the content. The adapted Thinking Healthy component was found to be complicated for mothers to follow and for the CHWs to deliver, specifically in group sessions compared to individual session as the participants were shy to disclose personal problems in group setting. This might be attributed to the lack of intensive training with CHWs on the abstract theme of the original Thinking Healthy approach, and a lack of focus on the behavioral activation process. Social norms on sharing of personal information and privacy issues might also be a concern inhibiting open discussion of such issues in a group. The duration required for the Thinking Healthy component was compromised in the intervention sessions to accommodate other contents of the integrated package: nutrition, antenatal care, and hands-on stimulation activities. The mental health impact of the Thinking Healthy component in this intervention was not assessed, but it has been assessed in other studies where there was one on one delivery of the adapted Thinking Healthy curriculum [27,28]. In addition, the behavior modification based on input from the mothers increases the potential for the success of this component, with a greater focus on the relatable character of the story. This adaptation also has the potential to reduce the sensitivity of sharing their own view on unproductive behaviors in group setting. Based on the qualitative findings, the Thinking Healthy component was further revised mainly focusing on behavioral activation in the symbolic story, made more participatory, and simplified. This adapted component is likely feasible to integrate with other intervention components and deliver by CHWs following a comprehensive training.

The strength of this study is that the integrated intervention extends the reach of a child development intervention by including pregnancy and post-childbirth periods, both critical periods for mothers and their young children. The pilot trial was successfully implemented by local women recruited as CHWs, which enhances the scope of scalability of this intervention. Engaging local women also has the potential to make them feel empowered and encourage other women in the community to participate and contribute to such developmental program. In addition, the study followed a systematic adaptation process which included incorporation of feedback from participants and intervention facilitators throughout the revision process, consistent with the process of Goldstein et al. [24,34]. These stages have also been applied in adaptation of an anger management intervention [35] and exposure therapy for the treatment of post-traumatic stress disorder [36]. In this paper we have described the intervention adaptation process. The later phases of the process mentioned by Goldstein et al. include conducting an initial open trial and randomized controlled trial of the revised manual, and assessing the impact of this integrated intervention. Aligned with these phases, a version of this intervention was tested in a subsequent randomized controlled trial with pregnant women and mothers of children under 24 months old (*n* = 621) in a rural setting and results are forthcoming [37].

The current study has some limitations. First, the small sample limits the scope for generalizability to other settings. However, the women in the sample have similar demographic characteristics to many other women in the area, and thus it is unlikely that the small sample size considerably affects the interpretation of this study. Second, the two-month duration is too short to examine any long-term barriers to session attendance. However, when asked about long-term attendance the majority of mothers indicated that they would be interested in attending sessions for one year, which indicates that mothers are motivated to attend.

## 5. Conclusions and Recommendations

The present study constitutes a systematic attempt to develop and implement an early child development intervention that includes an emphasis on pregnancy preparedness including regarding early interaction, basic nutritional information, and maternal mental wellbeing. The adapted intervention was acceptable by the participants and deliverable by the CHWs in both individual and group meetings. The revised curriculum addresses the feedback from the participants and CHWs to minimize the challenges faced throughout the process and has the flexibility to be delivered in individual or group sessions of 5–6 pregnant women and mother–child dyads. As we found similar type of adherence of mothers to both type of session delivery (group and combined), it is worth to design future trials with “group sessions” for cost effectiveness and scalability. Future work is recommended to evaluate the impact of this intervention on child development, maternal parenting behavior, and maternal mental health.

## Figures and Tables

**Table 1:** Adaptation phases of integrated intervention.

Study Phases	Goldstein et al.’s Manual Adaptation Process	Adaptation Stages of the Integrated Intervention in Parallel with the Process Developed by Goldstein et al.
Phase 1: Identify core components and structure of intervention	Stage-1: Choose a base manual for adaptation	The Reach-up curriculum for psychosocial stimulation was revised: contents for pregnancy and 0–5-month-old children were added.
	Stage-2: Conduct a focus group with the new target population	Two small group discussions with nine pregnant women and nine lactating mothers were used to identify the core contents of the intervention.
	Stage-3: Make initial manual revisions	The integrated components of the intervention curriculum were revised based on the results of two small group discussions.
Phase 2: Pilot initial revision of intervention	Stage-4: Pilot initial revisions of the manualized intervention	The adapted intervention package was piloted with 11 pregnant women and 29 mothers of children <24 months of age with two different delivery strategies.
Phase 3: Identify feedback from participants’ and CHWs	Stage-5: Conduct facilitator focus group	Interviews were conducted with the two facilitators and eight participants to identify areas for modification.
	Stage-6: Acquire expert review of the revised manual	The qualitative data from the pilot were systematically analyzed and reviewed by child development specialists to identify the barriers and facilitators of intervention delivery and uptake.
	Stage-7: Incorporate staff and expert feedback	The curriculum was revised incorporating feedback from the facilitators, participants, and child development specialists.

**Table 2:** Major findings of small group discussion with pregnant women and lactating mothers.

Major Theme	Pregnant Women	Lactating Mothers
General perception of factors affecting early childhood development	Emphasized the role of nutrition more than early care and interaction for child development.	Emphasized the role of nutrition for brain development.
Interaction with children	Limited knowledge about the impact of the surrounding environment on child development; No knowledge on age-specific play activities with children.	Lack of knowledge about the importance of early interactions with young children; Lack of awareness of the possibility of interactions with children during daily household activities.
Maternal mental health	Accepted that prenatal mood swings are an ordinary part of pregnancy, and no additional effort is needed for mental wellbeing; Understood the harmful impact of maternal mental health on child’s growth but did not know the ways to address this.	Lack of awareness regarding the importance of maternal mental health after childbirth; Acknowledgment of harsh disciplinary actions on children due to negative mood; Acknowledge that they did not have time for pleasurable activities due to a heavy workload after childbirth.
Pregnancy problems	Lack of awareness of danger signs during pregnancy.	NA
Negative thinking/Alternative beliefs	Self-blaming for inability to perform regular activities; Alternative understandings of self-care and diet during pregnancy prevailed in the community, leading to unfavorable attitudes toward pregnant women.	NA

**Table 3:** Demographic characteristics of intervention participants.

Characteristics (*n* = 36)	*n* (%)
**Types of participants**	
Pregnant women	9 (25)
Mothers of children <2 years old	27 (75)
**Mother’s education**	
Below primary or no formal education	6 (16.7)
Primary education	26 (72.2)
Secondary education	4 (11.1)
**Father’s education**	
Below primary or no formal education	13 (36.1)
Primary education	17 (47.2)
Secondary education	6 (16.7)
**Participants’ occupation**
Housewife	36 (100)
**Husbands’ occupation**
Irregular income (e.g., day labor, farming)	18 (50)
Salaried work (e.g., government or private job with fixed monthly salary)	6 (16.7)
Other (e.g., shopkeeper, retired, living abroad)	12 (33.3)
**Monthly Income**
<8000 BDT (<94 USD)	4 (11.1)
8000–20,000 BDT (94–235 USD)	29 (80.6)
>20,000 BDT (>235 USD)	3 (8.3)
**Housing construction (Wall material)**
Cement	7 (19.4)
Corrugated iron sheets	28 (77.8)
Mud	1 (2.8)

**Table 4:** Lessons learned and mitigation strategies.

Study Activities	Challenges	Mitigation Strategies
Initial curriculum development	Many knowledge gaps were identified among both pregnant and lactating women in the community.	Information about the most commonly reported knowledge gaps crucial for early child development were included in the curriculum.
Selection and training of CHWs	It was difficult to find CHW candidates who had completed secondary school because of the low prevalence of secondary school completion in the study area.	Emphasis was put on performance during interviews, as opposed to education to select candidates.
	There were social norms against women working outside the home, which made the four selected CHW candidates reluctant to attend the residential training.	One family member was permitted to accompany each trainee during the residential training.
	Some CHW candidates who performed well during the in-house training session did not perform well during the practice of session delivery with real mothers.	CHWs were selected based on the classroom performance, attitude, and field performance.
Pilot intervention	Some husbands and family members were not supportive of pregnant women and mothers attending sessions.	One-on-one motivational meetings were organized by the CHWs to encourage attendance.
	Some of the sessions lasted for more than 1 h because of the large amount of information delivered by CHWs, and sessions had little participatory engagement.	The curriculum content was restructured, emphasizing information for the specific age group present, and adding guidelines for interactive session delivery.
	The adapted Thinking Healthy approach was difficult for the CHWs to deliver in an interactive way.	A story telling approach was included, focusing more on behavioral activation in real life situations, e.g., practicing productive behavior leading to improved physical and mental health.
	The session providers found it difficult to identify and convey the key behavior recommendations presented in each session.	The session guidelines were adapted to include a summary with key points to deliver and the take home messages at the end of each session.

**Table 5:** Participants’ characteristics of qualitative interviews.

Respondents Code	Type of Respondents	Age (Years)	Last Completed Grade in School	Occupation	Monthly Household Income (BDT)
IDI-1	Pregnant woman (2nd trimester)	20	Grade 8	Housewife	6000/=
IDI-2	Lactating mother (8-month old child)	30	Grade 2	Housewife	5000/=
IDI-3	Pregnant woman (3rd trimester)	22	Grade 5	Housewife	6000/=
IDI-4	Lactating mother (6-month old child)	20	Grade-5	Housewife	20,000/=
IDI-5	Lactating mother (5-month old child)	24	Grade 6	Housewife	6500/=
IDI-6	Lactating mother (7-month old child)	20	Grade 7	Housewife	25,000/=
IDI-7	Pregnant woman (2nd trimester)	26	Grade 10	Housewife	6000/=
IDI-8	Pregnant woman (2nd trimester)	30	Grade 6	Housewife	15,000/=
KII-1	CHW	25	Higher secondary level	Bachelor’s degree student	-------
KII-2	CHW	18	Secondary level	Higher secondary school student	--------

**Table 6:** Thematic model of intervention facilitators and obstacles.

Theme	Sub-Theme	Core Findings
**Intervention content**		
	Facilitators to intervention success	Mothers were most interested in learning about health care and nutrition (7 out of 8); Interaction with children and play activities to promote early childhood development was a new idea for most of the mothers (5 out of 8; 4 lactating and 1 pregnant woman); Some behavior changes were identified: more ANC visits (2 out of 4 pregnant women), childcare activities (3 out of 4 lactating mothers), rest (4 out of 8), and nutritious food (5 out of 8) were reported by the pregnant women and lactating mothers.
	Obstacles to practice recommended behavior	Parenting advice was thought of as less important during the pregnancy period (3 out of 4); Conforming to some health care related recommendation was impeded by alternative beliefs (2 out of 4); The Thinking Healthy approach about identifying one’s own unhealthy thought patterns and replacing them with productive thoughts and behaviors was critical for mothers to understand (2 KII), and behavior modification by productive thinking was identified by less than half of the participants (3 out of 8).
	Suggestions for revision	Pregnant women were eager to learn more about pregnancy care and complications (3 out of 4); Different channels of communications (e.g., audio-visual strategies) for intervention delivery (4 out of 8).
**Intervention strategy and participation**		
	Facilitators to intervention success	The 40–50 min duration for group meetings seemed adequate for mothers (5 out of 8); Mothers were enthusiastic to participate for a longer duration during individual meetings (4 out of 8); Given the opportunity, mothers reported that they were willing to participate in such interventions for 1 year (7 out of 8); Assistance from family members in managing household chores and children sometimes encouraged mothers to participate in the sessions (6 out of 8).
	Obstacles to session delivery and participation	Pregnancy symptoms hindered session attentiveness (2 out of 4); Mothers were uncomfortable to discuss sensitive issues in group settings (1 KII); Expectations of financial incentives led to negative attitudes of family and neighbors toward the intervention (3 out of 8).
	Suggestion for revision	Participant’s husbands and neighbors would have a better understanding of the intervention if they were occasionally invited to participate in the sessions; this might enable more participation of pregnant women and mothers (4 out of 8).
